# Type 1 diabetes and low carbohydrate diets—Defining the degree of nutritional ketosis

**DOI:** 10.1111/dme.15178

**Published:** 2023-07-26

**Authors:** Hakan Ozoran, Michael Matheou, Pam Dyson, Fredrik Karpe, Garry D. Tan

**Affiliations:** ^1^ Oxford Centre for Diabetes, Endocrinology and Metabolism University of Oxford Oxford UK; ^2^ Oxford Centre for Diabetes, Endocrinology and Metabolism Oxford University Hospitals Foundation Trust Oxford UK; ^3^ NIHR Biomedical Research Centre Oxford University Hospitals Foundation Trust Oxford UK

**Keywords:** carbohydrate metabolism, ketoacidosis, ketone measurement, type 1 diabetes

## Abstract

**Aims:**

Adopting a low‐ or very low‐carbohydrate (LCD or VLCD) diet in type 1 diabetes mellitus (T1D) is a controversial intervention. The main fear is that these diets may increase the risk of diabetic ketoacidosis. However, there is little data about the ketoacidosis risk and the level of physiological nutritional ketosis in individuals following these diets. We aimed to define the level of ketosis in those with T1D following carbohydrate restricted diets in a real‐world observational study.

**Methods:**

Patients with T1D who had self‐selected dietary carbohydrate restriction were enrolled from local clinics and were compared to those following an unrestricted regular carbohydrate control diet (RCCD). Participants completed a 3‐day diary, documenting food intake, ketones, and blood/interstitial glucose concentrations.

**Results:**

Participants were divided into three groups according to mean carbohydrate intake: VLCD (<50 g carbohydrates/day) *n* = 6, LCD (50–130 g carbohydrates/day) *n* = 6, and RCCD (>130 g carbohydrates/day) *n* = 3. Mean beta‐hydroxybutyrate (BOHB) concentrations were 1.2 mmol/l (SD 0.14), 0.3 mmol/l (SD 0.12) and 0.1mmol/l (SD 0.05) in the VLCD, LCD and RCCD groups, respectively (*p* = 0.02). Post hoc Dunn test demonstrated this reached statistical significance between the VLCD and RCCD groups (*p* = 0.02).

**Conclusion:**

Carbohydrate restricted diets, in particular VLCDs, are associated with a higher BOHB level. However, the degree of ketosis seen is much lower than we expected, and significantly lower than the level typically associated with diabetic ketoacidosis. This may suggest the risk of ketoacidosis is lower than feared, although safety will need to be evaluated further in large scale randomised trials.

## Graphical abstract

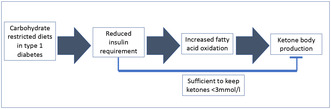

**What's new?**

- Dietary carbohydrate restriction has become an increasingly popular, and contentious, approach selected by patients living with type 1 diabetes.
- A key question that remains is whether adherence to a low‐ or very low‐ carbohydrate diet in type 1 diabetes is associated with a dangerous, supraphysiological ketonaemia?
- Carbohydrate restriction may offer therapeutic benefit and a much‐needed alternative approach to the management of type 1 diabetes.

Adopting a low‐ or very low‐carbohydrate (LCD or VLCD) diet in type 1 diabetes mellitus (T1D) is a controversial intervention. Proponents of LCD/VLCDs report improvements in glycaemic control, decreased glycaemic variability and insulin requirements. However, the use of LCD/VLCDs by people with T1D is not recommended by professional organisations, mainly due to paucity of adequately controlled studies to examine their efficacy and safety. There is concern that physiological nutritional ketosis seen in LCD/VLCDs may translate into an increased risk of pathological ketoacidosis.^1^ However, there is little data about the ketoacidosis risk with LCD/VLCDs^2^ and self‐selected groups of people with T1D following these diets report that ketoacidosis is rare.^3^ Furthermore, the level of nutritional ketosis observed in individuals with T1D following an LCD/VLCD is poorly described, with inconsistent measurements of ketone concentrations at infrequent time points.^4^, ^5^, ^6^, ^7^, ^8^

To better define the diurnal changes in nutritional ketosis in T1D, we performed a real‐world observational study in a single tertiary diabetes centre in England, comparing ketone levels in those following an LCD/VLCD to those following an unrestricted carbohydrate counting diet. Adult participants with T1D of at least 3 years duration, who had either self‐selected an LCD/VLCD or were following an unrestricted regular carbohydrate control diet (RCCD) were enrolled from outpatient diabetes clinics at the Oxford Centre for Diabetes, Endocrinology and Metabolism from May to September 2022. Baseline anthropometric data, daily insulin use, HbA1c, GOLD score and history of admissions with ketoacidosis or severe hypoglycaemia in the preceding 2 years were collected from self‐reported questionnaires and electronic patient records. Repetitive ANOVA test with post hoc Tukey HSD was used to assess for between group differences in these characteristics. Participants completed a 3‐day diary, documenting diurnal capillary ketone and blood/interstitial glucose concentrations using home glucose and ketone meters at the following time points: fasting, pre‐meal, 2‐h post meals and pre‐bedtime. Participants also completed a food diary, including weights and photographs of food where possible, which was subsequently analysed with nutritics software to define macronutrient composition. Participants were divided into three groups according to their mean carbohydrate intake: VLCD (*n* = 6), LCD (*n* = 6) and RCCD (*n* = 3). Mean diurnal BOHB (beta‐hydroxy butyrate) concentrations for each individual was calculated using an area under the curve (AUC) method. The non‐parametric independent samples Kruskal‐Wallis test and post‐hoc Dunn's test were used to test for significance.

The mean daily carbohydrate intakes in the groups were 26 g (VLCD, range 15–36 g), 57 g (LCD, range 37–93 g) and 256 g (RCCD, range 232–269 g). There were no statistically significant differences between the groups in age, total daily insulin dose, BMI or GOLD score. There were no reported admissions for ketoacidosis or severe hypoglycaemia in the preceding 2 years in any participants in the groups. The mean diurnal BOHB concentrations were 1.2 mmol/L (SD 0.14), 0.3 mmol/L (SD 0.12) and 0.1 mmol/L (SD 0.05) in the VLCD, LCD and RCCD groups, respectively (*p* = 0.02). A post‐hoc Dunn test revealed that this difference reached statistical significance between the VLCD and RCCD groups (*p* = 0.02) but not between the VLCD and LCD (*p* = 0.06) or LCD and RCCD groups (*p* = 0.56) (Figure 1). The range of mean diurnal BOHB was 0.6–1.15 mmol/L for VLCD, and 0.3–0.7 mmol/L for LCD groups and 0.1–0.2 mmol/L for RCCD groups.

**FIGURE 1:**
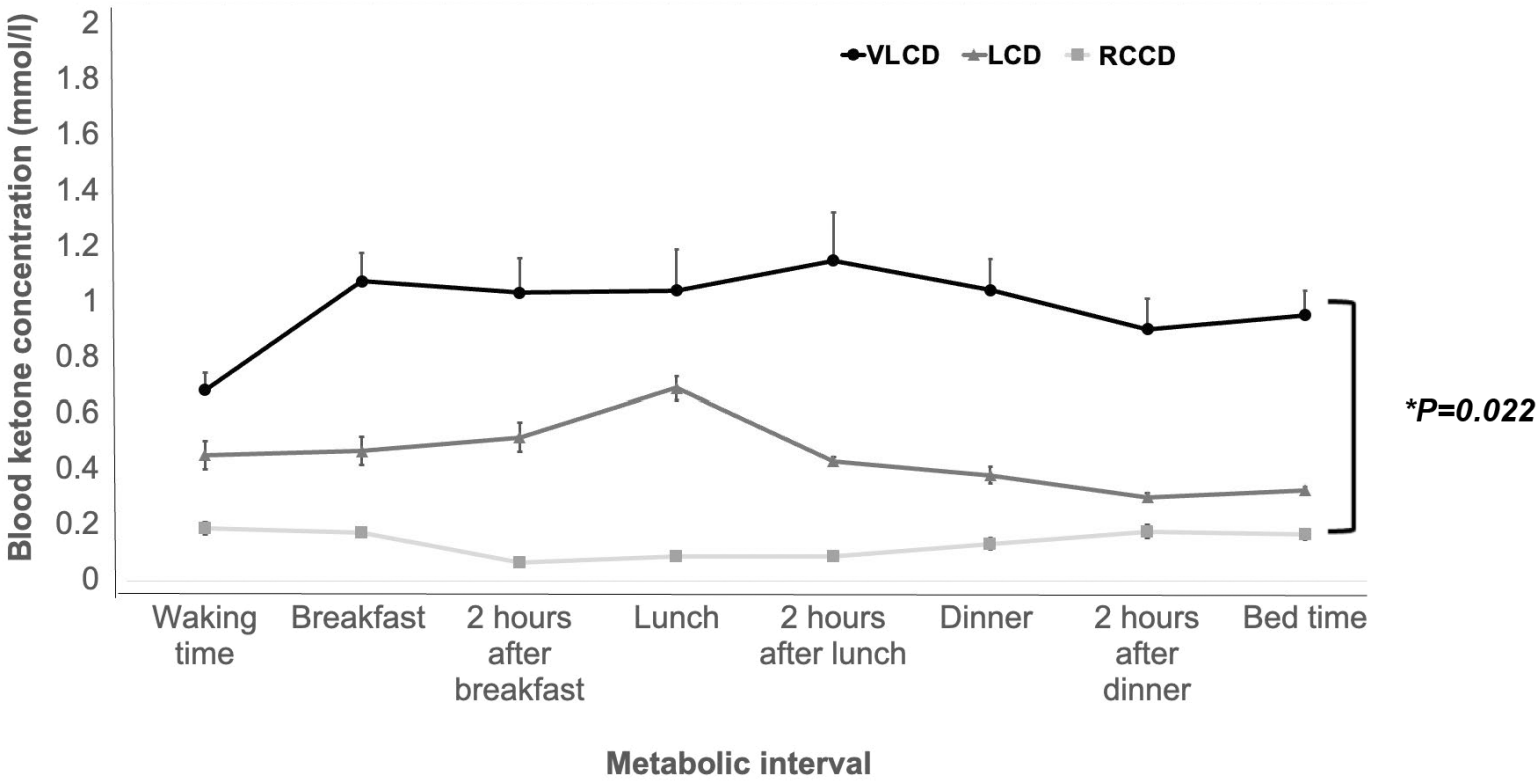
Mean diurnal capillary beta‐hydroxy butyrate (BOHB) concentration at different metabolic intervals for each group. Black circles = very low‐carbohydrate (VLCD), grey triangle = low‐carbohydrate (LCD) and grey square = regular carbohydrate control diet (RCCD). Error bars correspond to standard error.

Expectedly, there was a negative relationship between BOHB and mean daily carbohydrate intake. This relationship is non‐linear, and there appears to be a point of inflection below 30 g carbohydrate a day, where further reductions in CHO intake are associated with a sharp rise in BOHB levels.

Carbohydrate restricted diets, in particular VLCDs, are associated with a higher capillary concentration of BOHB compared with unrestricted carbohydrate diets. However, despite severe carbohydrate restriction, the degree of nutritional ketosis seen is much lower than expected, with a range of 0.3–1.15 mmol/L in the carbohydrate restricted groups. This is significantly lower than the threshold of 3 mmol/L that is commonly associated with diabetic ketoacidosis. Although it is difficult to extrapolate about the risk of ketoacidosis from our small observational study, the low concentrations of BOHB and seemingly long‐term stable adaptation to carbohydrate restriction seen in our cohort may suggest that the risk is lower than feared. The possibility of a sharp rise in ketosis below 30 g carbohydrate per day is an interesting observation which warrants further confirmation in larger studies, as this may provide an important clinical threshold to help advise patients of a carbohydrate level below which risk of ketoacidosis may increase significantly. Large scale randomised trials further evaluating the safety and efficacy of carbohydrate restriction as a dietary strategy in type 1 diabetes are needed. Given that only 8.1% of individuals with type 1 diabetes achieved the recommended HbA1c of <48 mmol/L (<6.5%) in 2019/2020,^9^ should the safety of LCD/VLCDs be established, this may open an additional therapeutic option for individuals with type 1 diabetes in pursuit of glycaemic targets.

## AUTHOR CONTRIBUTIONS

H.O. and F.K. researched data, contributed to discussion and wrote, reviewed and edited the manuscript. M.M. reviewed and edited the manuscript. P.D provided diet and nutrient evaluations and both G.T. and P.D. contributed to discussion and reviewed and edited the manuscript. All authors approved the final version of the manuscript.

## CONFLICT OF INTEREST STATEMENT

The views expressed are those of the authors and not necessarily those of the NHS, the NIHR or the Department of Health.

## GUARANTOR STATEMENT

Hakan Ozoran and Fredrik Karpe are the guarantors of this work and, as such, had full access to all the data in the study and take responsibility for the integrity of the data and the accuracy of the data analysis.

## FUNDING INFORMATION

FK and GT are supported by the National institute for Health Reasearch (NIHR) Oxford. FK, GT and PD are also supported by the Biomedical Research Centre (BRC). FK is supported by the BHF RG17/1/32663.
